# Author Correction: Cell-type-specific and disease-associated expression quantitative trait loci in the human lung

**DOI:** 10.1038/s41588-024-01765-z

**Published:** 2024-04-25

**Authors:** Heini M. Natri, Christina B. Del Azodi, Lance Peter, Chase J. Taylor, Sagrika Chugh, Robert Kendle, Mei-i Chung, David K. Flaherty, Brittany K. Matlock, Carla L. Calvi, Timothy S. Blackwell, Lorraine B. Ware, Matthew Bacchetta, Rajat Walia, Ciara M. Shaver, Jonathan A. Kropski, Davis J. McCarthy, Nicholas E. Banovich

**Affiliations:** 1 Translational Genomics Research Institute, Phoenix, AZ USA; 2 St. Vincent’s Institute of Medical Research, Melbourne, Victoria Australia; 3 Melbourne Integrative Genomics, University of Melbourne, Melbourne, Victoria Australia; 4 Division of Allergy, Pulmonary and Critical Care Medicine, Department of Medicine, Vanderbilt University Medical Center, Nashville, TN USA; 5 School of Mathematics and Statistics, Faculty of Science, University of Melbourne, Melbourne, Victoria Australia; 6 Flow Cytometry Shared Resource, Vanderbilt University Medical Center, Nashville, TN USA; 7 Department of Cell and Developmental Biology, Vanderbilt University, Nashville, TN USA; 8 Department of Veterans Affairs Medical Center, Nashville, TN USA; 9 Department of Pathology, Microbiology and Immunology, Vanderbilt University Medical Center, Nashville, TN USA; 10 Department of Cardiac Surgery, Vanderbilt University Medical Center, Nashville, TN USA; 11 Department of Thoracic Disease and Transplantation, Norton Thoracic Institute, Phoenix, AZ USA

**Keywords:** Gene expression, Gene expression profiling, Gene regulation

Correction to: *Nature Genetics* 10.1038/s41588-024-01702-0, published online 28 March 2024.

In the version of the article initially published, in the legend to Fig. 6, the text now reading “Numbers of SNPs that were nominally significant (*P* < 1 × 10^−6^) in the IPF GWAS meta-analysis and also eQTL” originally read “Heatmaps presenting the numbers of eQTLs that were nominally significant (*P* < 1 × 10^−6^) in the IPF GWAS meta-analysis” and has now been corrected in the HTML and PDF versions of the article.

