# Supplementary material for: Cell-type-specific and disease-associated expression quantitative trait loci in the human lung
Source: Nat Genet. 2024 Mar 28;56(4):595–604. doi: 10.1038/s41588-024-01702-0 (PMC11018522; doi:10.1038/s41588-024-01702-0)
Supplement: Supplementary file 1 — Supplementary Figs. 1–24 and Notes 1 and 2. [file 41588_2024_1702_MOESM1_ESM.pdf]

nature genetics

Article https://doi.org/10.1038/s41588-024-01702-0

Cell-type-specific and disease-associated expression quantitative trait loci in the human lung

In the format provided by the authors and unedited

Supplementary Information

Supplementary Figures 1–24
Supplementary Note 1
Supplementary Note 2

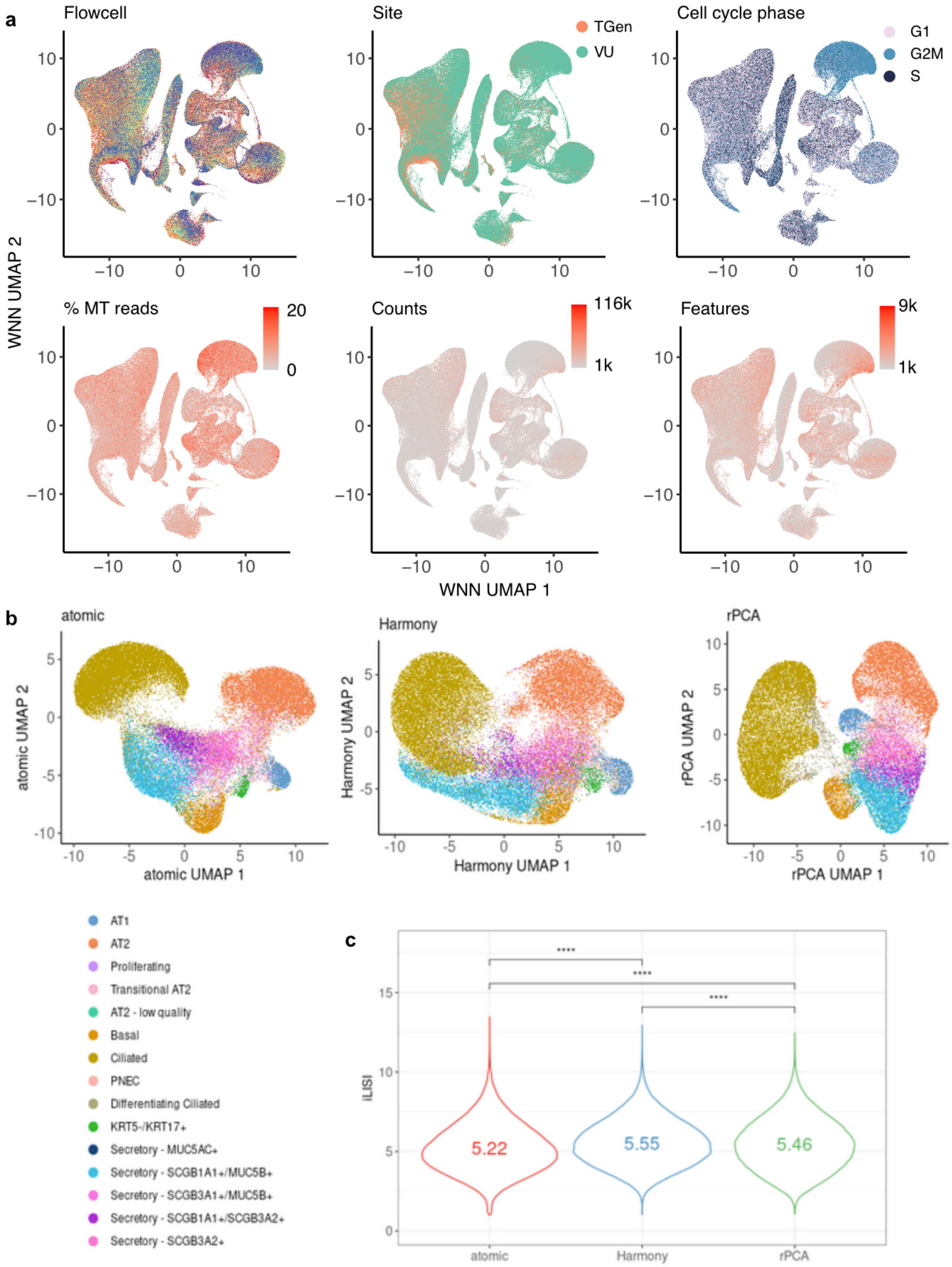

Supplementary Figure 1: a, UMAP dimensionality reductions of cells included in the pseudobulking and eQTL mapping, pseudocolored by flowcell, processing site (TGen or Vanderbilt), cell cycle phase, proportions of mitochondrial reads, number of read counts, and number of features. **b,** Comparison of three integration methods across the epithelial cell types.

c, iLISI for batch mixing with the three integration methods. **** indicates non-adjusted two-sided t-test $p \leq 0.0001$.

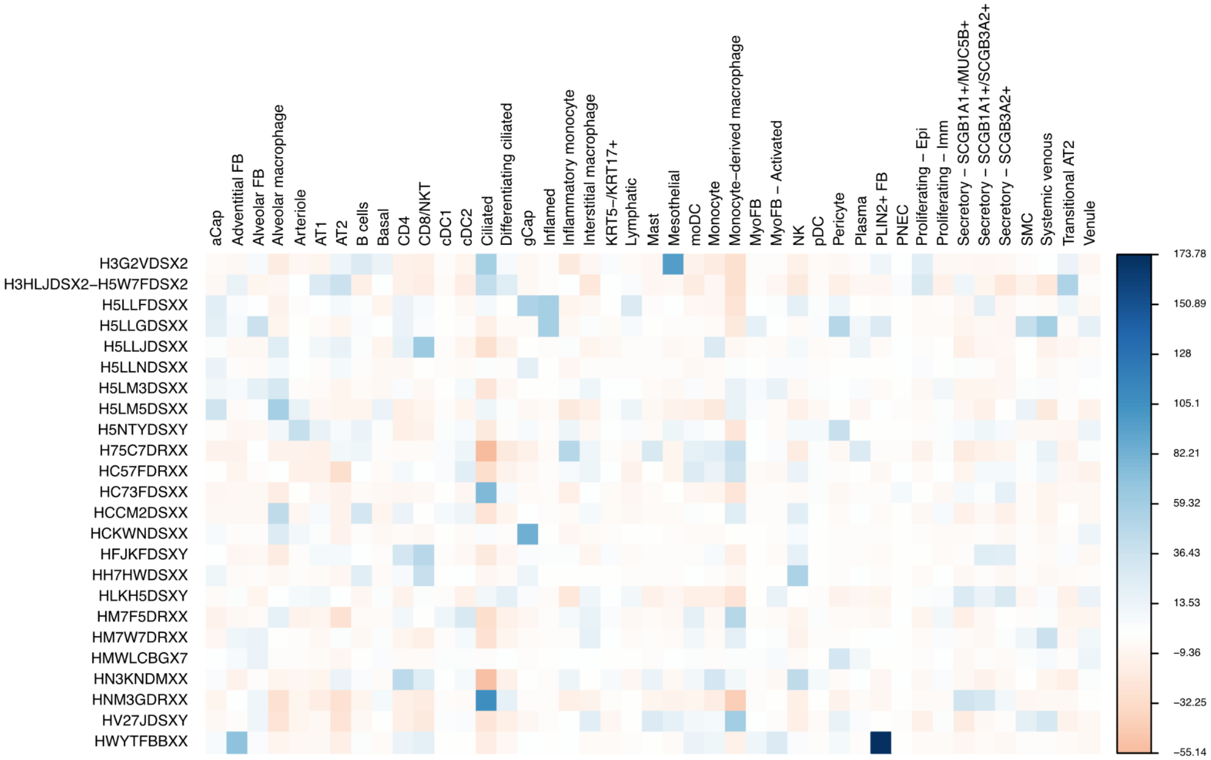

Supplementary Figure 2: χ^2 residuals of the observed and expected proportions of cell types across batches.

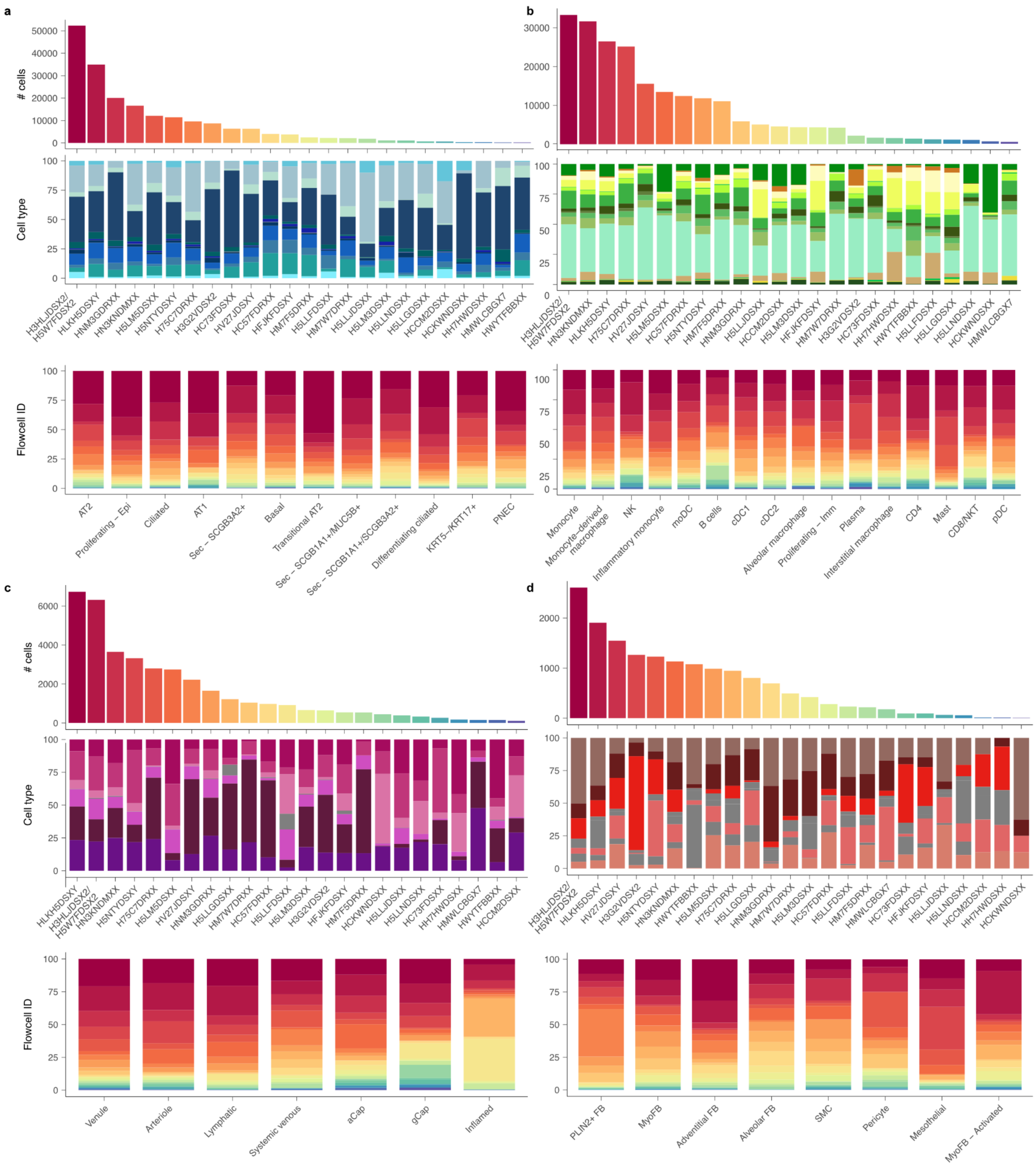

Supplementary Figure 3: Numbers of cells in each batch, proportions of cell types in each batch, and proportions of cells from each batch for each cell type in the (a) epithelial, (b) immune, (c) endothelial, and (d) mesenchymal populations.

Supplementary Note 1

Selecting optimal sample and cell number thresholds for pseudo-bulk eQTL mapping is challenging in the absence of ground truth. Here, drawing on the best practices presented in Cuomo et al.[1], we selected the inclusion criteria to maximize our ability to map eQTL in confidence across many cell types. Specifically, we included only samples with at least 5 cells for a given cell type. This threshold was selected to be loose enough to minimize donor loss, while still eliminating donors with poor expression support, as demonstrated by simulations and benchmarking by Cuomo et al.

This selection threshold aims to balance the trade-off of power and noise in pseudobulk eQTL analysis: a higher cell number threshold per pseudobulk profile produces less noisy profiles as they average over more single cells, but results in loss of cell types for analysis (e.g., if they have too few donors for inclusion) and loss of power in remaining cell types due to loss of donors/individuals and thus reduced sample size for eQTL mapping in some cell types. A more lenient threshold retains more cell types for analysis, getting a fuller picture of genetic regulation of gene expression throughout the tissue, and maximizes eQTL detection power. However, it can produce noisier pseudobulk expression profiles, possibly resulting in false positives or negatives.

A threshold of 5 cells per sample for a given cell type was previously used by Cuomo et al. in their simulation studies and detailed benchmarking of single-cell eQTL mapping results against those derived from matched bulk RNA-seq data. When exploring the effects of the simulated distributions of the cell numbers allocated for each sample and metrics such as power, empirical FDR, and beta correlation of the number of donors and the average number of cells per donor, Cuomo et al. demonstrate that the empirical FDR is consistent (~0.07) across values for average number of cells per sample (Cuomo et al. Supplementary Figure 7b). Examining the distributions of cells per donor in Cuomo et al. and this study (below), we find that the distributions are comparable, and thus, the simulation results are directly relevant to the present study. Thus, we expect our eQTL mapping results to not be enriched for false positives with the threshold of 5 cells. Using a more stringent threshold would result in a loss of samples available for eQTL mapping, with a substantial impact on discovery power and empirical FDR (50 vs. 87 donors in Cuomo et al. Supplementary Figure 7a). Thus, we have aimed to maximize the number of donors available for analysis to bring the empirical FDR as close as possible to the nominal FDR.

Distributions of numbers of cells allocated for each sample in simulations with an average of 50 or 120 cells, and the distribution of cells per donor per cell type in this study.

	Min	1st Qu	Median	Mean	3rd Qu	Max
Ave. 50 cells	8.00	24.25	44.00	48.68	67.00	157
Ave. 120 cells	23.00	59.25	113.50	123.46	157.75	481.00
This study	13.16	48.62	80.98	127.11	122.98	801.57

With the threshold of 5 cells per sample, all cell types included in the analysis had a minimum of 42 samples, 75% of cell types had at least 56 samples, and 50% had at least 76 samples. Comparing this distribution to Supplementary Figure 7 in Cuomo et al., we can confidently maximize detection power and control FDR.

We further evaluated the impacts of the minimum cell number on the number of available donors and cell types to be included in the eQTL analysis. As lung is a complex tissue with many distinct cell types, we endeavor to maximize the inclusion of as many cell types, as far as is reasonable, to gain as full a picture as possible of the landscape of genetic regulation of gene expression in healthy and diseased lungs.

Setting a threshold of at least 40 donors to include a cell type in eQTL mapping and downstream analyses, with the threshold we used (>=5 cells) 38 cell types were available for eQTL mapping. With a threshold of 10, 20, 30, or 50 cells, 30, 25, 21, and 16 cell types are retained, respectively. With a threshold of at least 50 cells per donor per cell type, we would only be able to map eQTL in 30-50% of major cell types in the lung. Using a threshold of at least 5 cells per donor allows us to map eQTL for 38 cell types, almost complete coverage of the major lung cell types.

Number of cell types with at least n samples meeting the minimum cell number threshold for n = 30, 40, 50, 60, 70

n_cell_threshold	n30	n40	n50	n60	n70
>=5_cells	40	38	32	28	27
>=10_cells	34	30	28	22	16
>=20_cells	29	25	21	15	7
>=30_cells	25	21	16	8	6
>=50_cells	21	16	8	5	4

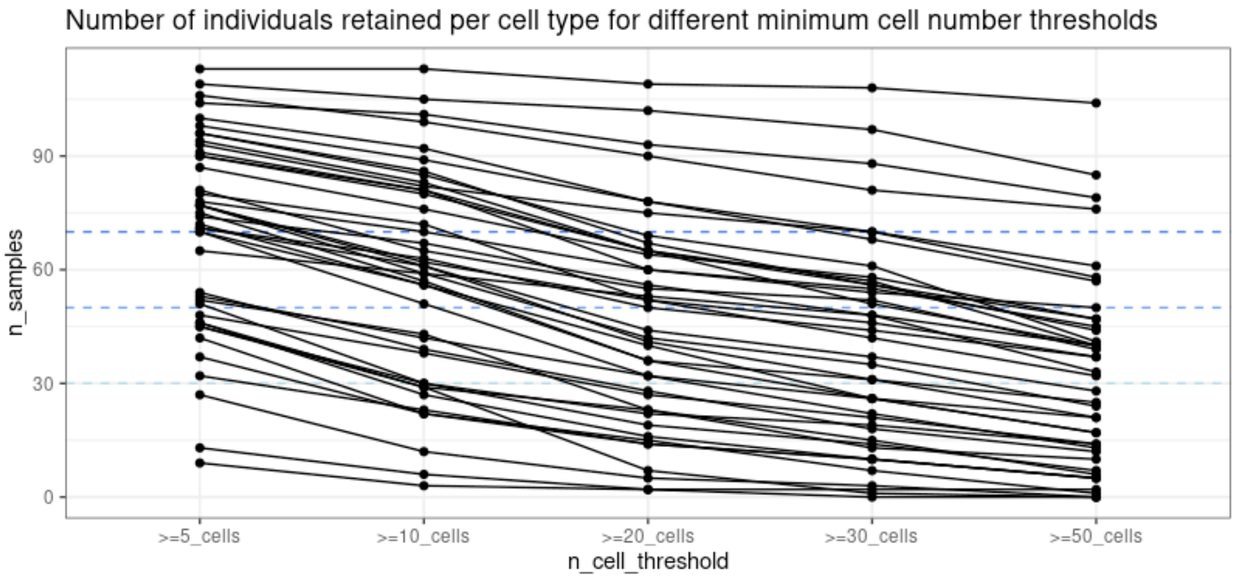

Finally, because the *mashr* joint modeling approach requires a complete eQTL matrix (i.e., no missing estimates for any eQTL for any cell type), eQTL mapping is carried out on some genes in certain cell types where there is relatively low power to detect eQTL effects. The application of *mashr* itself, by modeling covariance and effect sharing between cell types, drastically improves eQTL detection power even in underpowered cell types. Further, after applying *mashr*, a stricter inclusion filter is applied where mashr-adjusted eQTL effects are only reported for genes meeting the expression criteria for that given cell type. Thus, on balance, the use of *mashr* supports a minimum cell threshold of 5 cells to include as many cell types in the analysis as possible and, simultaneously, using *mashr* mitigates against potential issues that might otherwise arise in eQTL mapping from cell types with smaller sample sizes and therefore lower power. Our ability to account for FDR is demonstrated by the permutations (**Supplementary Figure 7-8**).

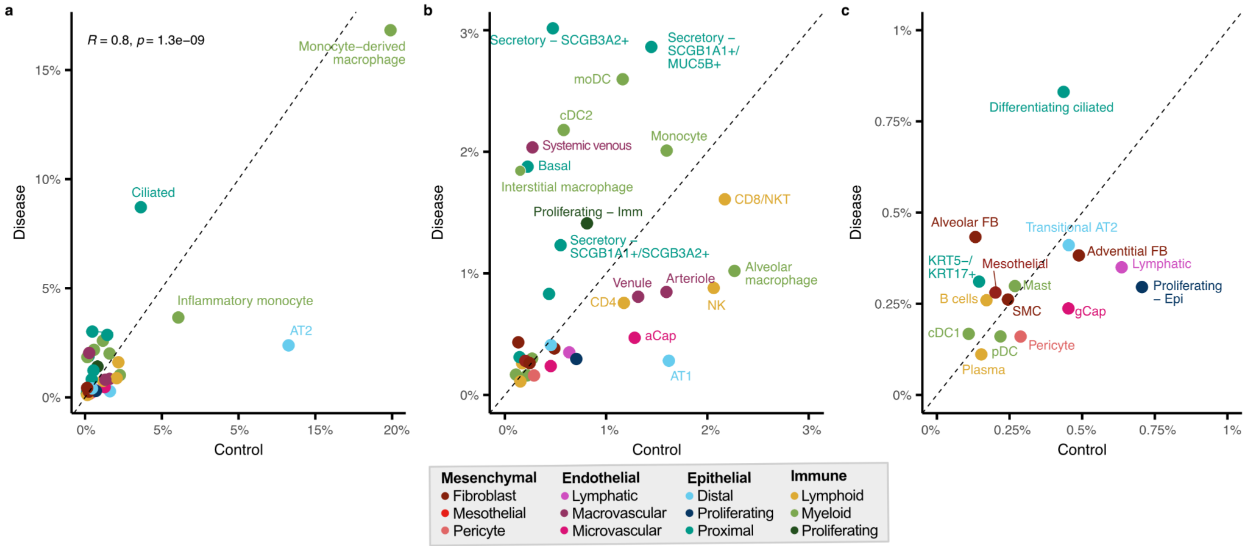

Supplementary Figure 4: Comparison of the median cell type abundance between control (x-axis) and diseased (y-axis) individuals for cell types included in eQTL mapping (# cell types = 38). The dashed line represents equal abundance in control and diseased samples. Cell types are colored by sub-lineage. **a,** All cell types shown with cell types with >3% abundance labeled. Pearson's correlation shown. **b,** Includes cell types with <3% abundance, with cell types with >1% abundance labeled. **c,** Includes and labels all cell types with <1% abundance.

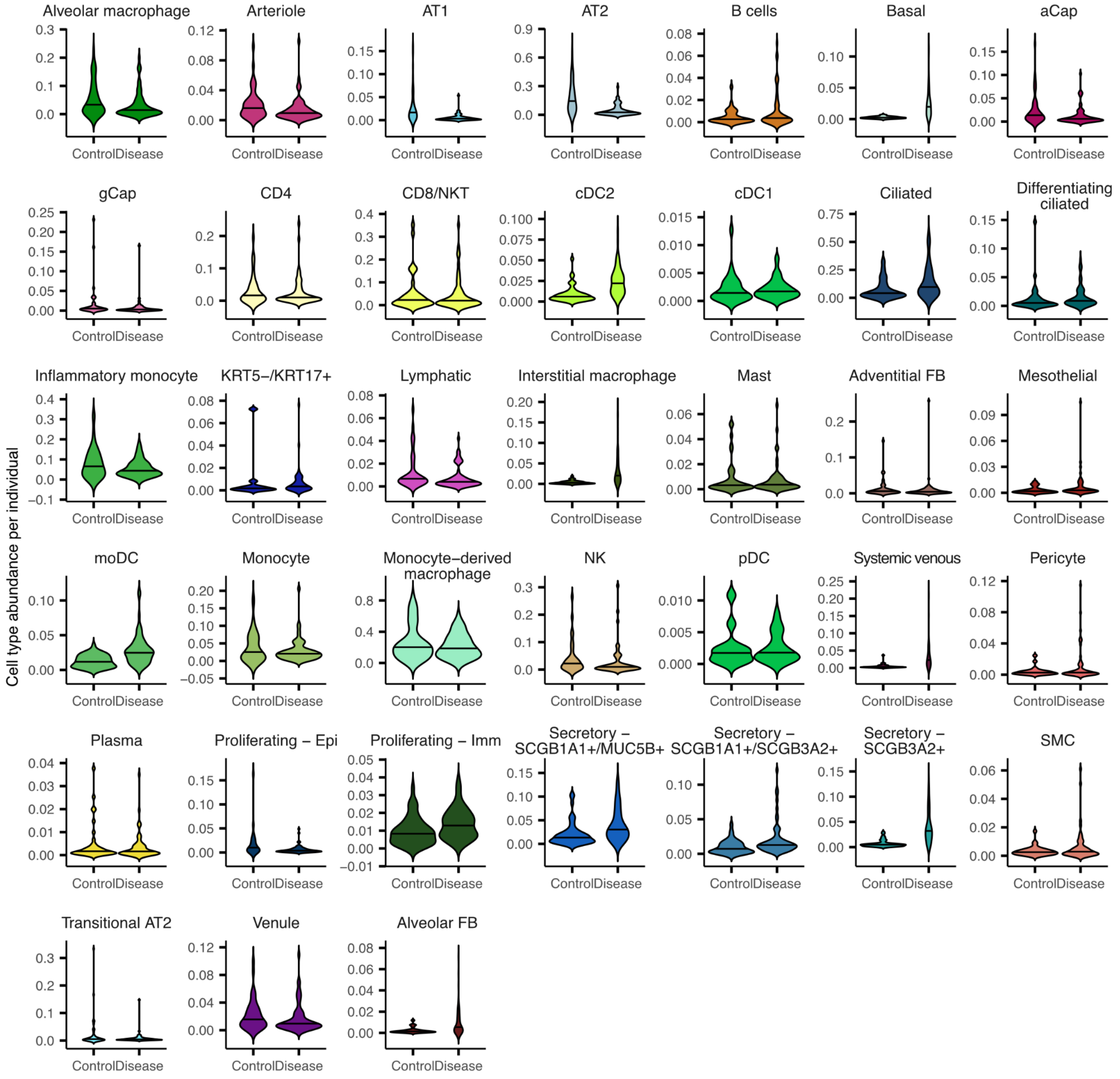

Supplementary Figure 5: The distribution of cell type abundance per individual grouped by disease status (control, n=48, vs. diseased, n=68). The bar represents the median abundance.

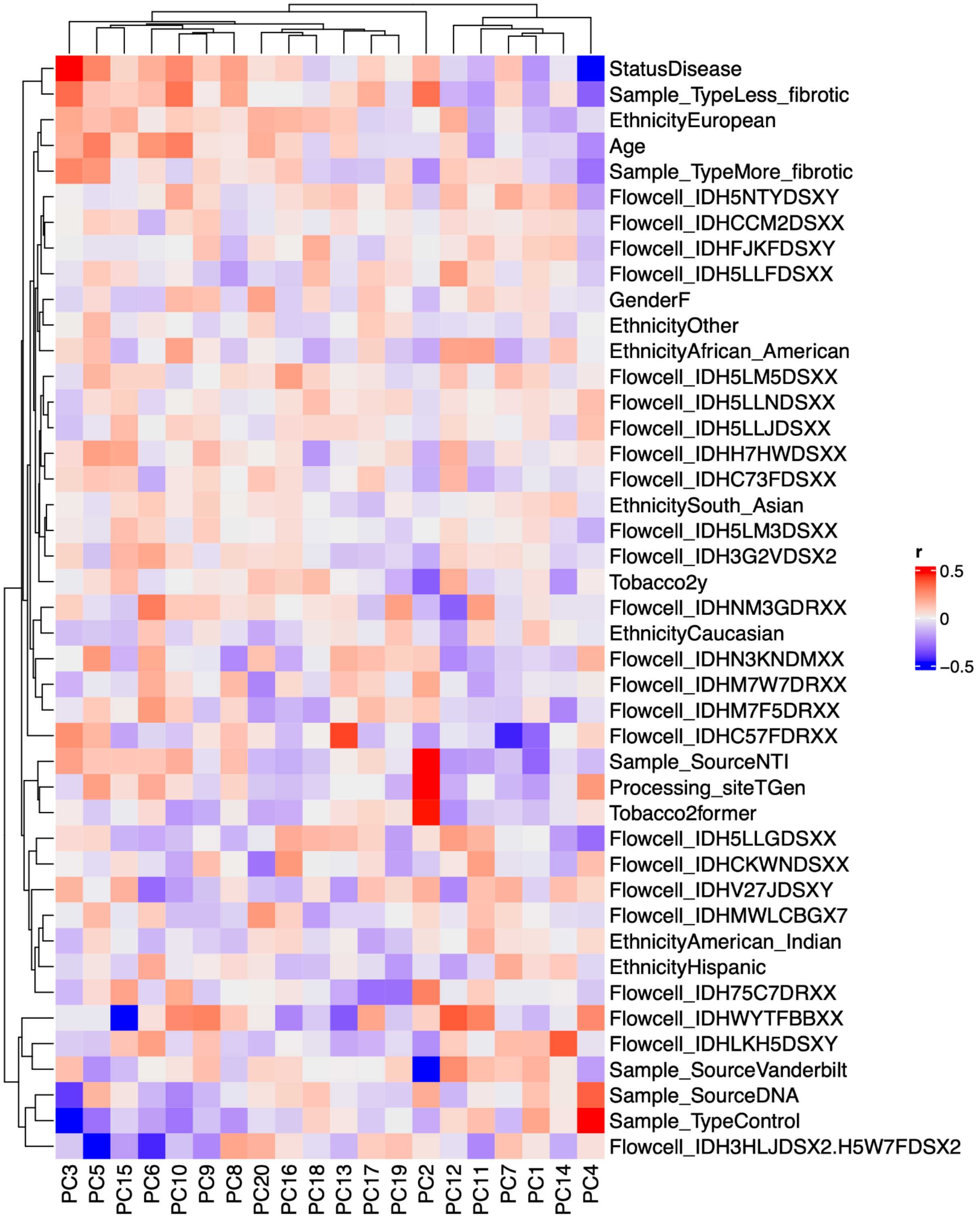

Supplementary Figure 6: Heatmap of correlations between the PCs included as covariates in the eQTL analysis of AT2 cells and known covariates.

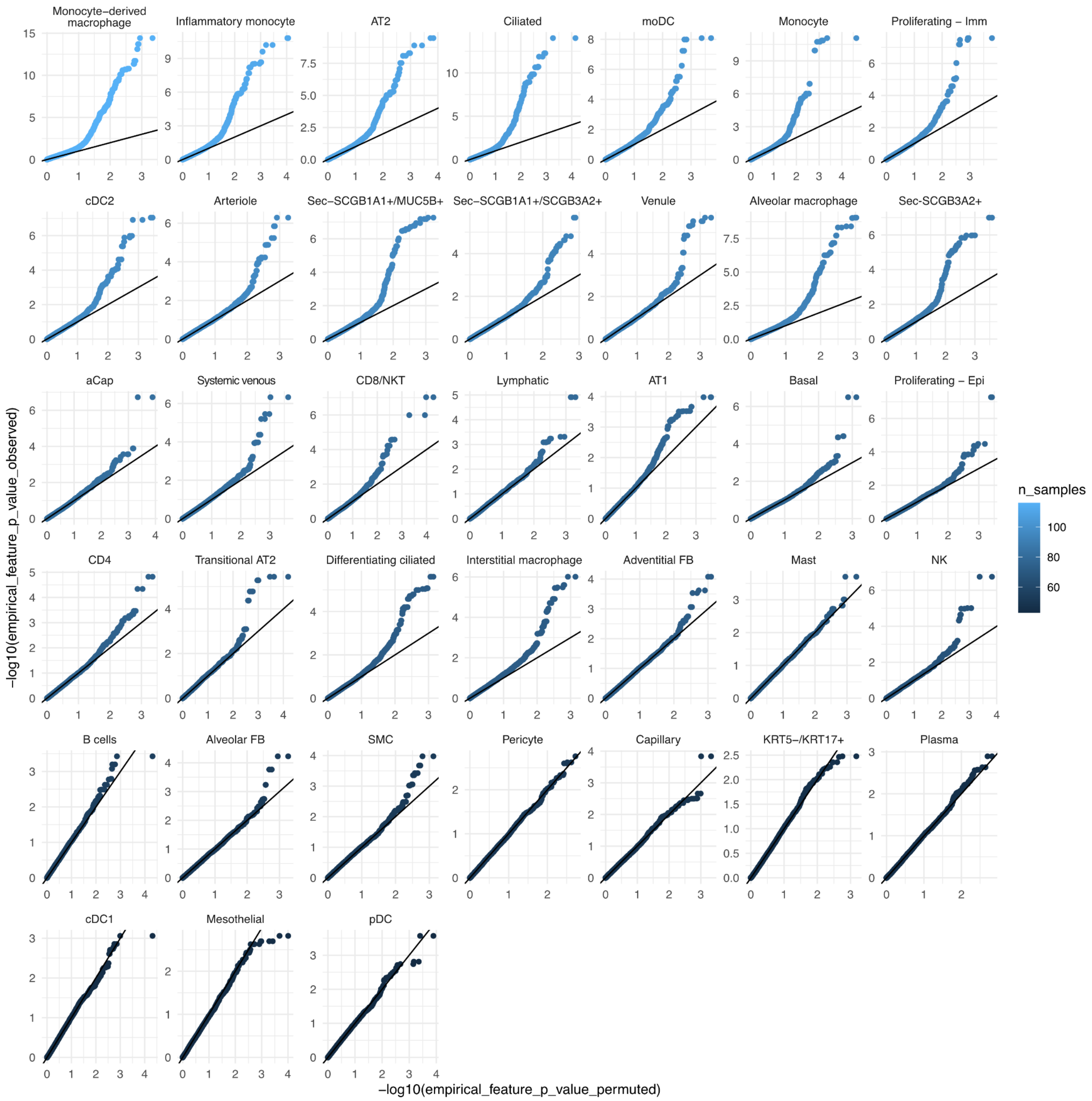

Supplementary Figure 7: Quantile-quantile plots for each cell-type showing the observed empirical p-values of the top hit per gene (y-axis) against the permutation-based (genotypes were shuffled independently for each cell-type) empirical p-values of the top hit per gene (x-axis). Empirical p-values are from the limix sc-eQTL mapping runs and are shown on the -log10 scale. Observed and permuted values were sorted from largest to smallest.

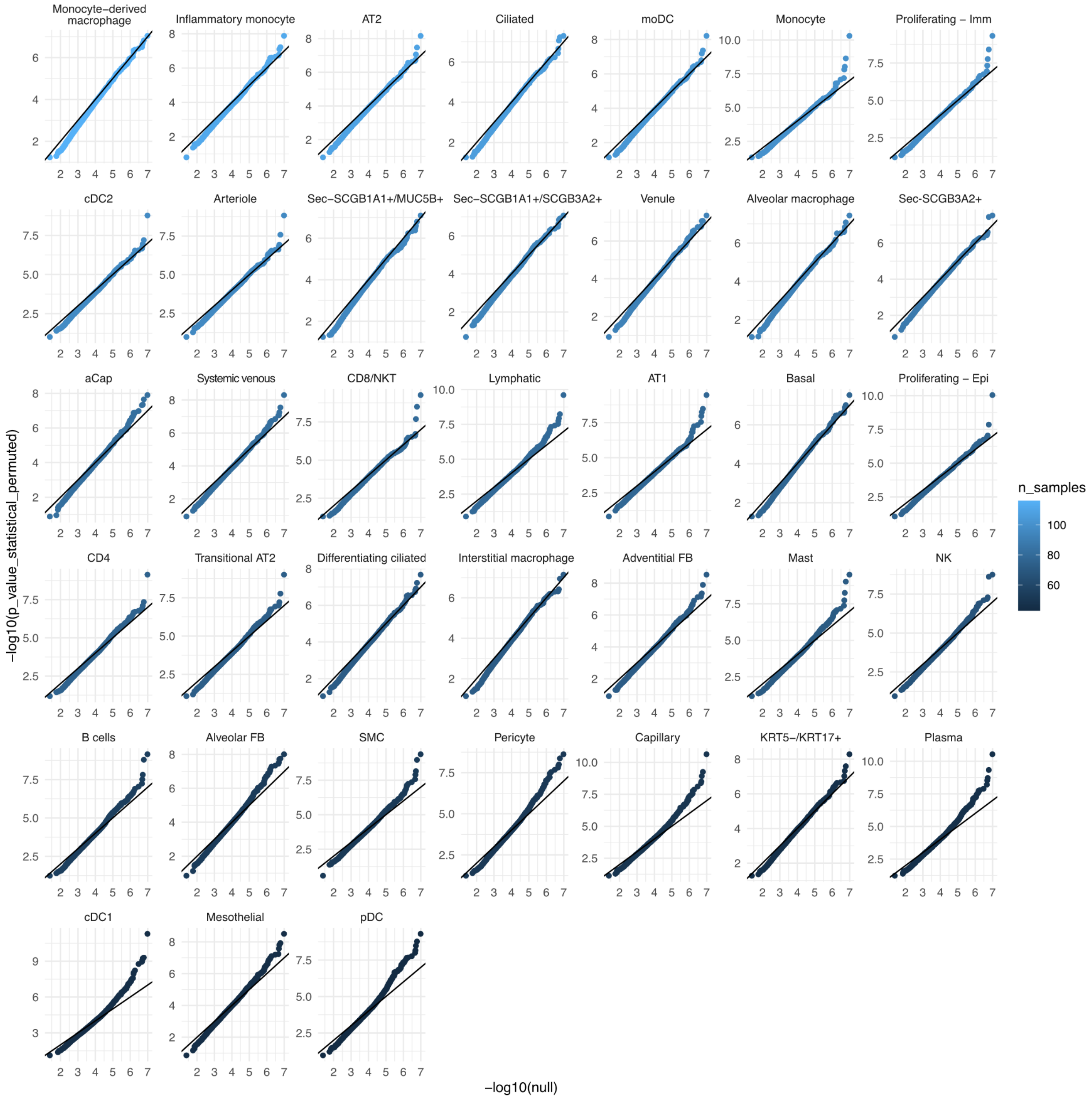

Supplementary Figure 8: QQ plots for each cell-type showing the expected statistical *p*-values under the null hypothesis (x-axis) against the permutation-based statistical *p*-values using limix to run sc-eQTL mapping with permuted genotypes (y-axis). The null hypothesis was generated by, for each gene, taking the minimum value after sampling N from a uniform distribution (min=0; max=1), where N is the number of SNPs tested for that gene.

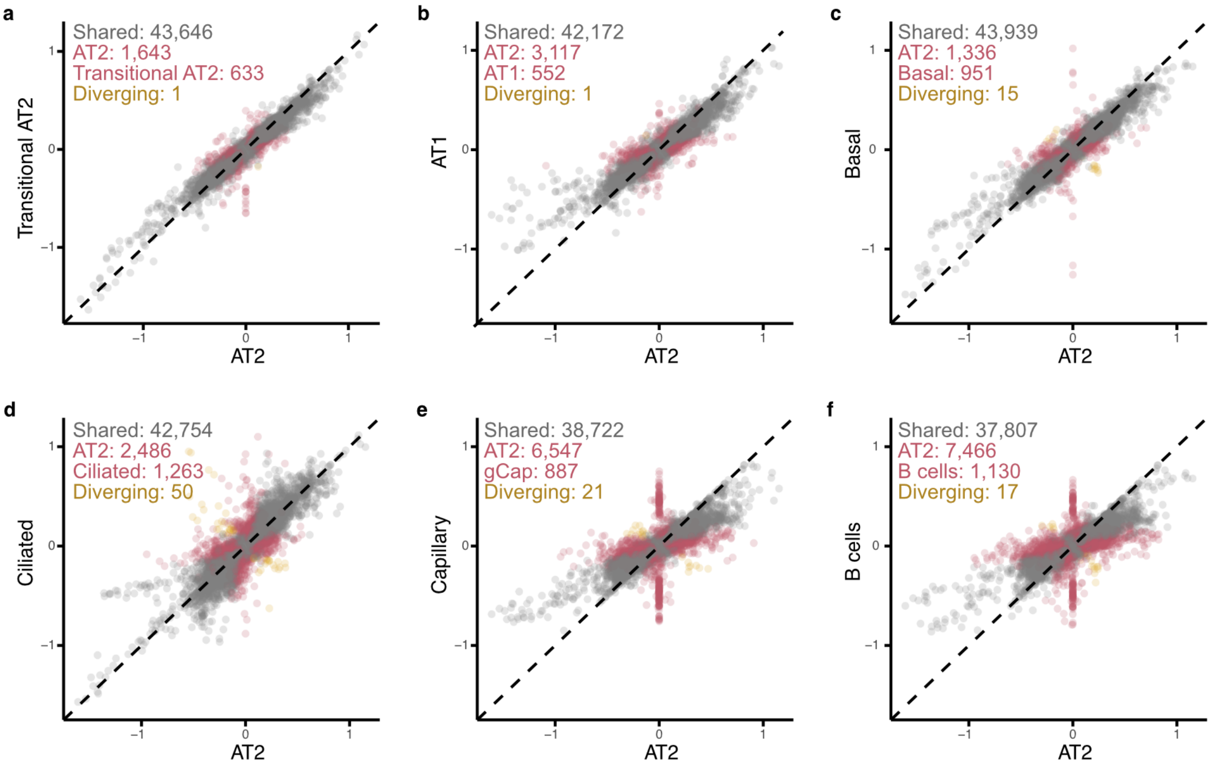

Supplementary Figure 9: Comparison of mashr estimated effect sizes for top eQTL between AT2 and other cell types. The number of top eQTL that are significant and in the same direction (shared), significant only in AT2, significant only in the comparison cell type, or significant in both but with an opposite direction of effect (diverging) are reported.

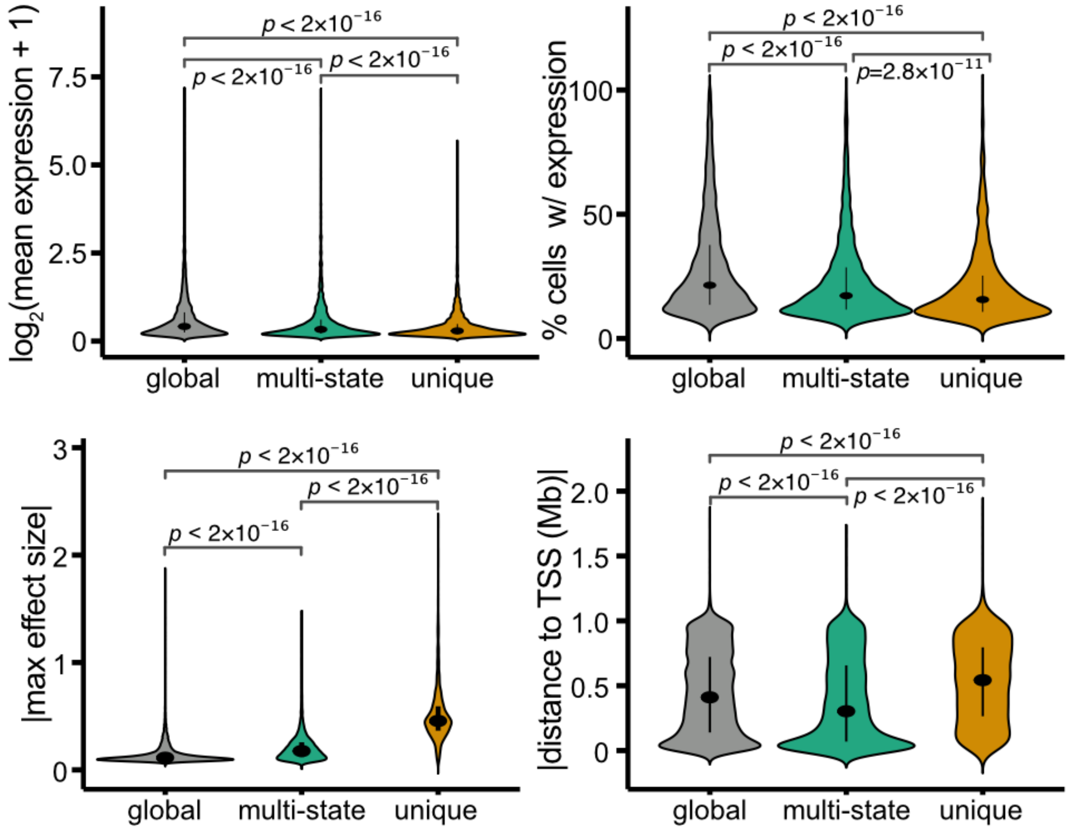

Supplementary Figure 10: Mean normalized expression (first panel) and percentage proportion of cells expressing (second panel) the target eGenes of eQTL unique to a single cell type (n=2,358), shared across multiple cell types (n=21,793), or globally shared across all cell types (n=26,355), as well as the absolute effect sizes (third panel) and absolute distances to the target eGene TSS (fourth panel). In violinplots, sample median and the 25th and 75th percentiles are indicated. Non-adjusted two-sided t-test *p*-values are indicated.

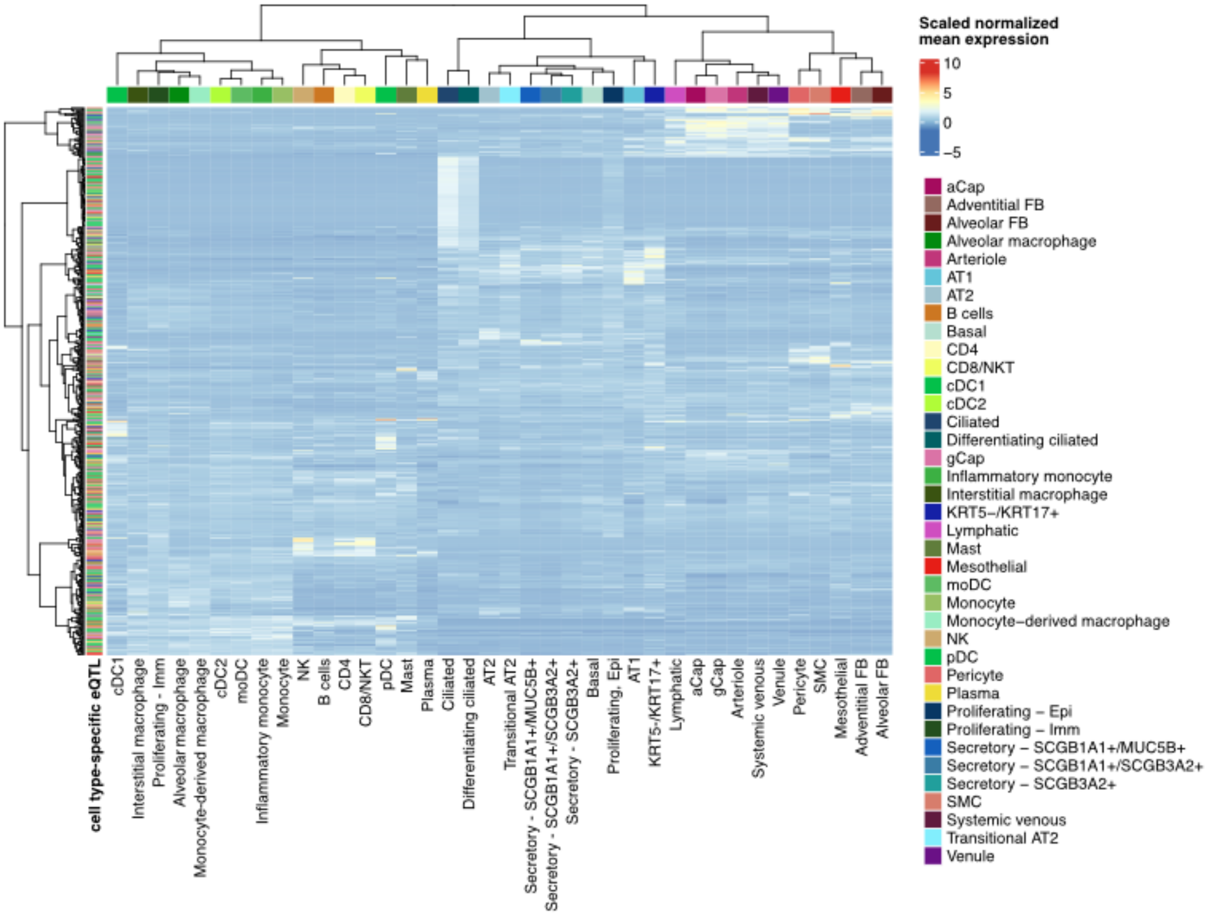

Supplementary Figure 11: Expression of eGenes unique to a single cell type (n=584).

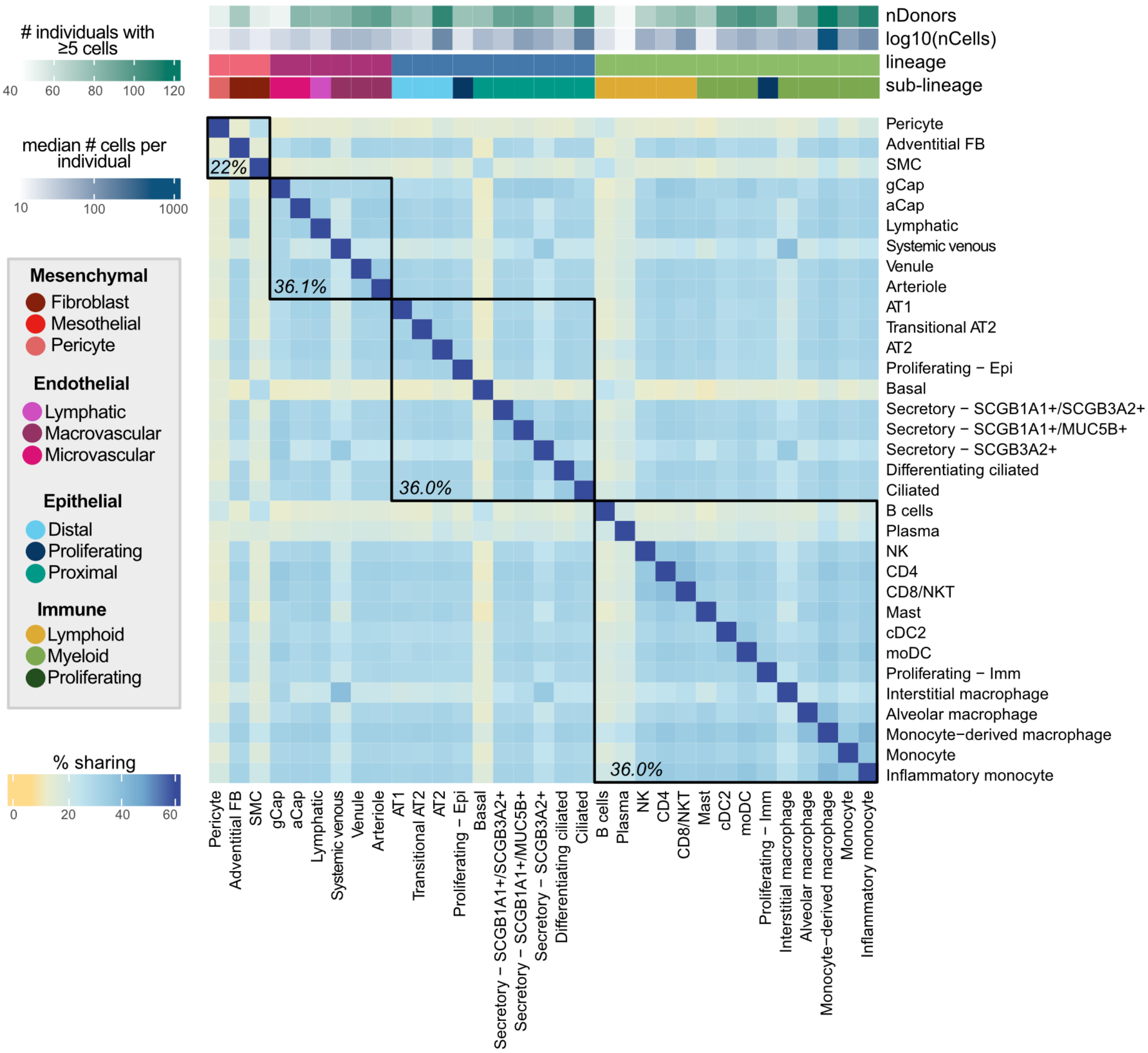

Supplementary Figure 12: Percent of top int-eQTL that are shared between two cell types. Top eQTL are considered shared if they are significant in both cell types (local false sign rate ≤ 0.1) and the mashr estimated effect size is within a factor of 0.5. Cell types are annotated above by lineage, sublineage, the number of individuals with ≥5 cells, and the median number of cells per individual for that cell type. Median pairwise percent sharing per lineage is shown in black.

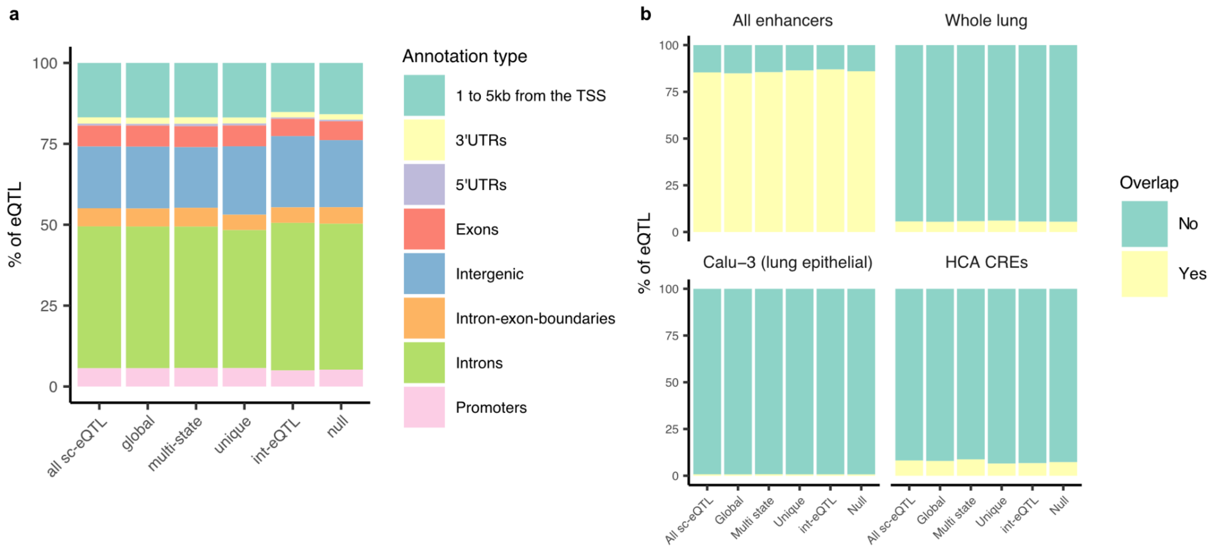

Supplementary Figure 13. Cell type-eQTL, int-eQTL, and a null set of non-eQTL SNPs annotated for genic regions and among all enhancers in EnhancerAtlas 2.0, lung tissue enhancers, and human lung epithelial cell line (Calu-3) enhancers, as well as the *cis*-regulatory elements in the Human Cell Atlas. The set of non-eQTL SNPs was selected to match the total number of significant sc-eQTL and their distribution of distances to target gene transcription start sites.

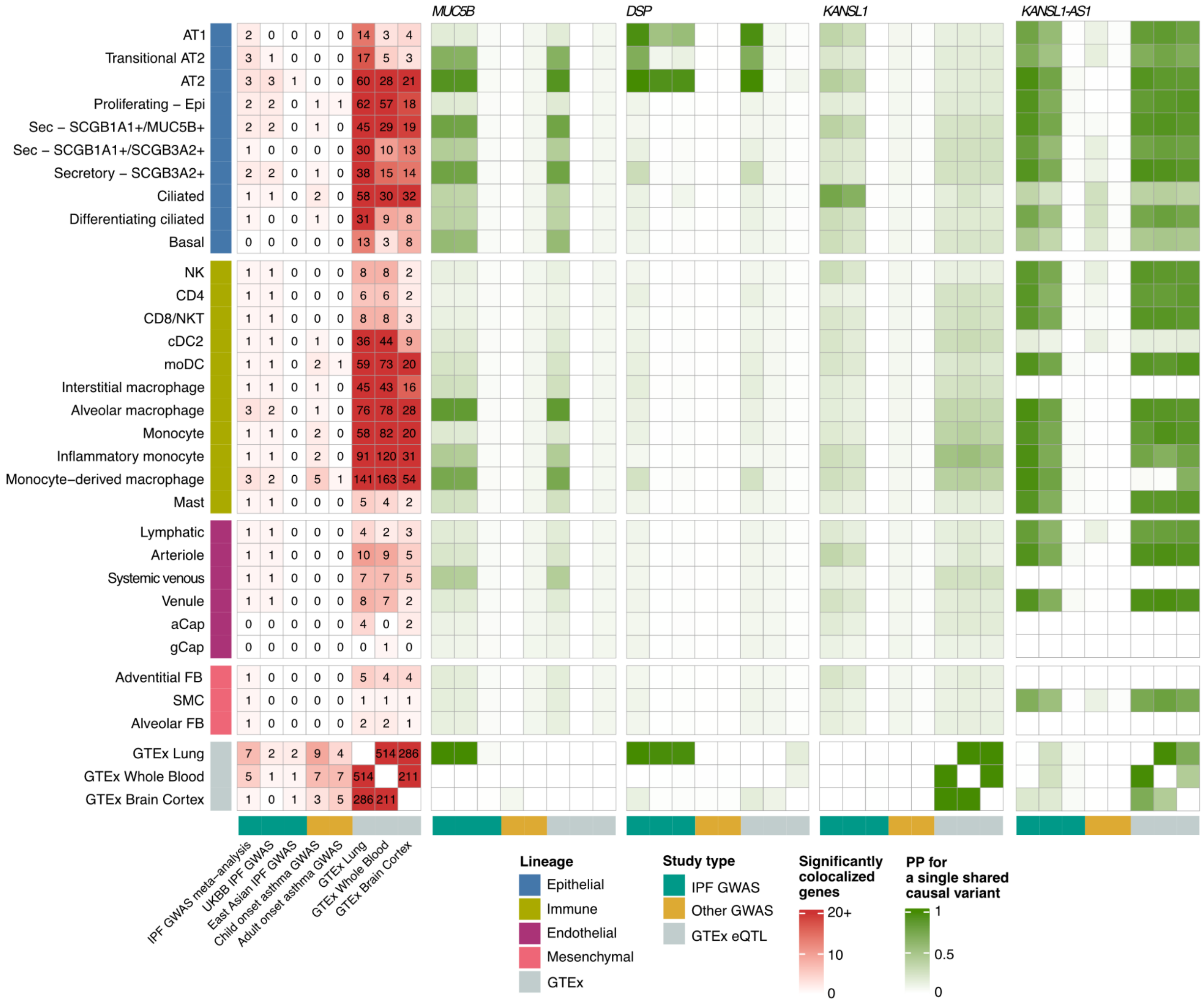

Supplementary Figure 14: Patterns of colocalization between cell type-eQTL, bulk-eQTL, and lung trait GWAS. The left-most heatmap presents the numbers of significantly colocalized genes between the 38 cell types and three GTEx tissues, three IPF GWASs, and child and adult-onset asthma GWAS. The green-shaded heatmaps present posterior probabilities for a single shared causal variant between the tested cell types and GWAS for top IPF GWAS-implicated genes (*MUC5B*, *DSP*, *KANSL1*, *KANSL1-AS1*).

Supplementary Note 2: Replication of cell type-eQTL among GTEx and PBMC cell type-eQTL.

To better contextualize the replication of the eQTL identified in this study, we compared our results to previous bulk and single cell results (**Supplementary Figure 15**). Focusing first on the colocalization analysis presented in the manuscript (Fig. 6) between cell type eQTL and bulk eQTL from GTEx whole blood, lung, and brain, we found the highest degree of colocalization colocalization between Monocyte-derived macrophages and GTEx whole blood (8.55%). Given that Monocyte-derived macrophages were one of the most abundant cell types this is perhaps expected. However, we found overall, immune cell type eQTL tend to have the highest levels of colocalization with GTEx whole blood, followed by lung, and then by brain. Conversely, non-immune cell types tend to have the highest colocalization with GTEx lung, followed by whole blood, then by brain. These results suggest our cell type level eQTL are capturing relevant tissue-specific signals. Further, we find the effect sizes of cell type and bulk-eQTL to be highly correlated between well-powered and closely related cell types/tissues, with an R^2 of 0.318 between AT2 cells and GTEx lung. In contrast, comparisons between lower abundance cell types and non-lung bulk-eQTL resulted in the lowest correlations, with an R^2 of 0.0883 between cDC1 cells and brain cortex.

While colocalization analysis represents the most stringent approach for intersecting genomics associations, we can also identify shared associations by assessing eQTL that are significant in both studies. To this end, we find that all classes of eQTL are significantly enriched among GTEx lung eQTL, with 21.9% of multi-state, 19.1% of globally shared, 11.7% of eQTL unique to a single cell type, and 13.4% of int-eQTL replicating in GTEx lung (**Fig. 5f,** GTEx nominal $p<1\times10^{-6}$). We explored the level of top-eQTL overlap with GTEx on a cell-type level. Up to roughly 10% of cell type-eQTL replicate in GTEx, with the highest overlap (10.37%) between Inflammatory monocytes and whole blood eQTL and the lowest (0.79%) between Alveolar fibroblasts and brain cortex. Across cell types, the highest level of overlap was with GTEx whole blood, likely due to the higher sample size. All three approaches, and the colocalization and effect size correlation, in particular, reveal a lineage-specific pattern of overlap that reflects the expected similarity of cell types and tissues included in the analysis.

To contextualize these results compared to prior single cell-eQTL studies, we examined cell type eQTL from 6 PBMC cell types (n=982) reported in another study.[2] This study did not carry out colocalization analysis but found 40.4% of cell type-eQTL replicated among GTEx whole blood.[2] When employing the same significance threshold as Yazar et al., we find that 12.6% of our immune cell type-eQTL replicate among GTEx blood, and 11.6% of all cell type-eQTL in our analysis replicate in GTEx lung. Further, using the threshold employed by Yazar et al., 36.3%, 28.5%, and 38.3% of the GTEx lung, whole blood, and brain cortex eQTL were significant eQTL in at least one of the cell types in our study. We further compared the eQTL detected by Yazar et al. to our cell type-eQTL. Out of the 848 eQTL for NK cells and 104 eQTL for plasma cells detected by Yazar et al. that were also tested for in our study, 31.0% and 19.2% were significant in our analysis of these cell types, respectively.

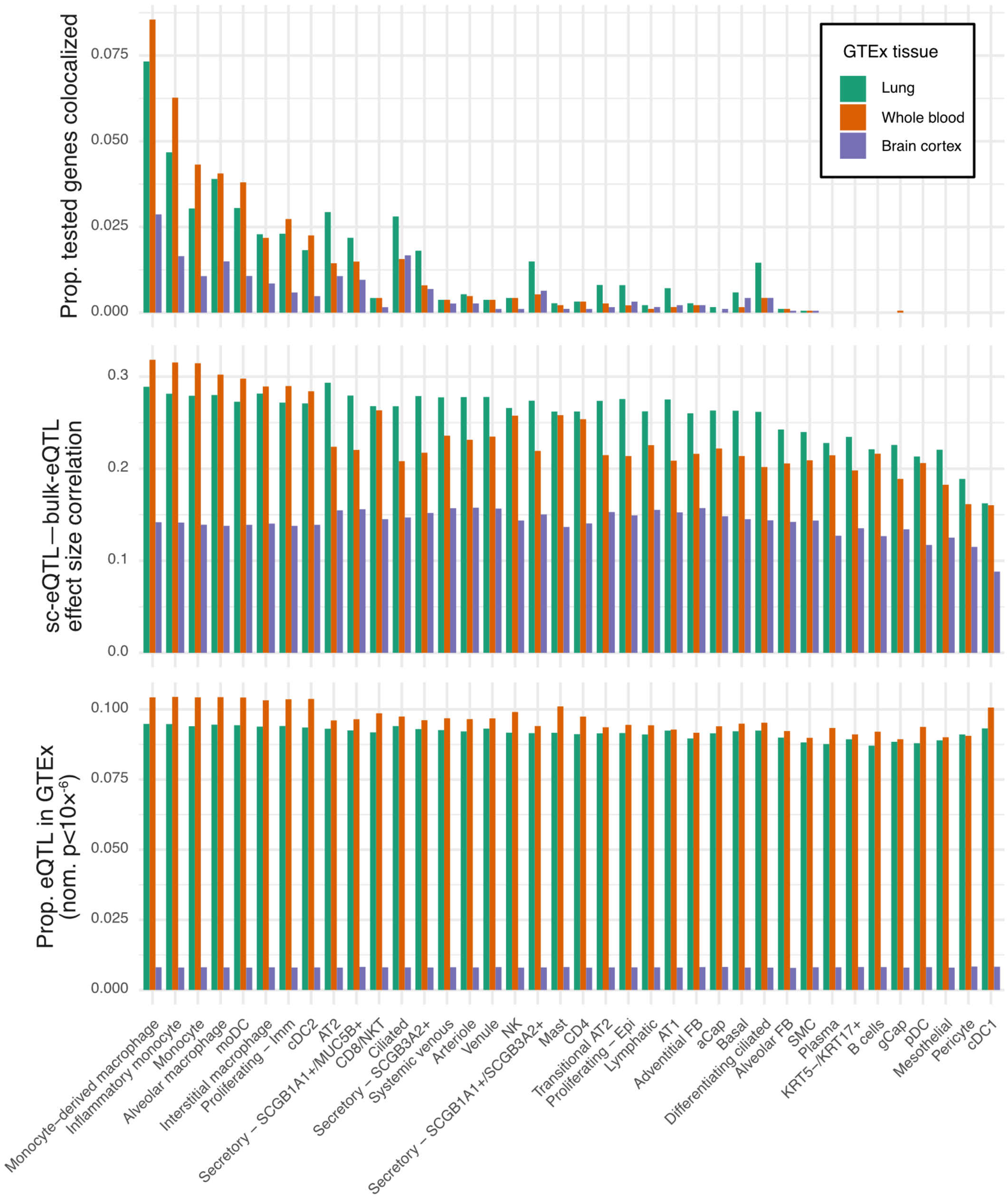

Supplementary Figure 15: For each cell type, the proportion of the tested genes that colocalized with GTEx bulk-eQTL (top), the correlation of eQTL effect sizes (R^2) between cell type-eQTL and GTEx bulk eQTL (middle), and the proportion of eQTL that replicate in GTEx with a nominal $p<1\times10^{-6}$ (bottom).

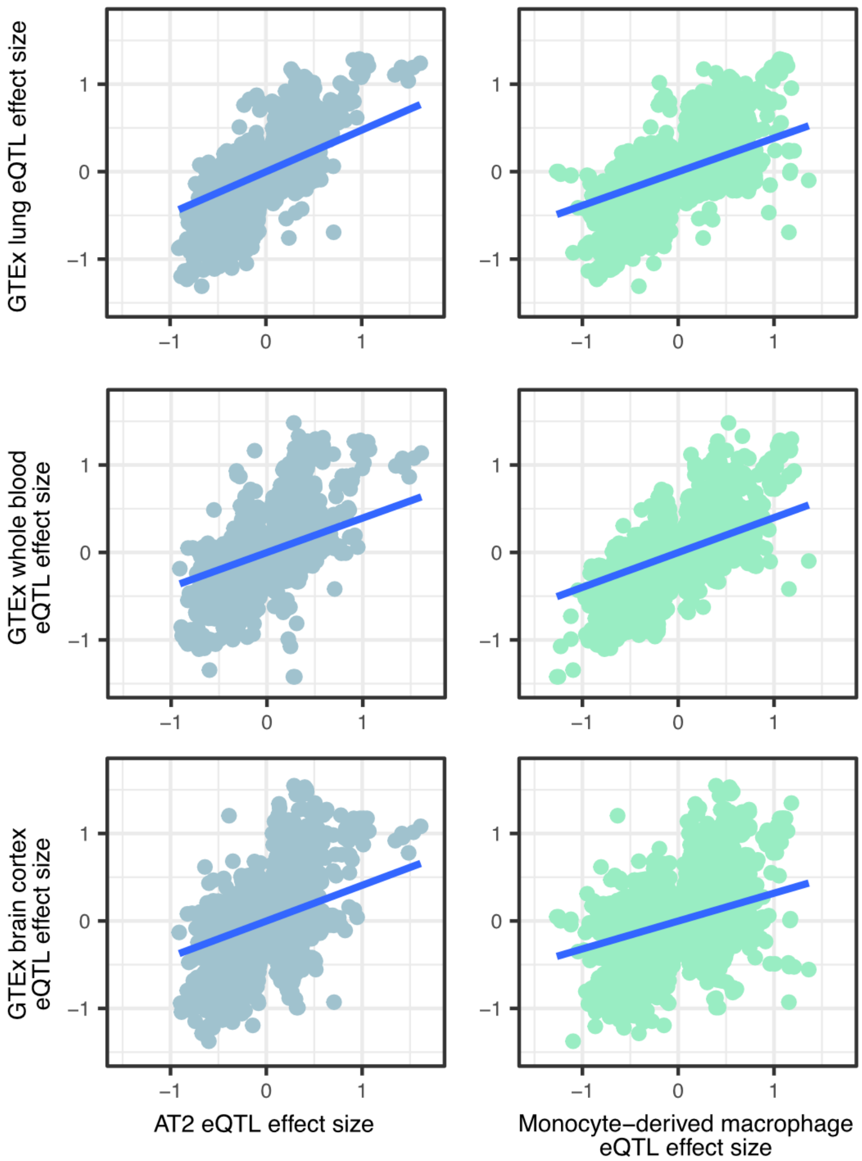

Supplementary Figure 16: eQTL effect sizes from GTEx lung, whole blood, and brain cortex (y-axis) and epithelial AT2 cells and monocyte-derived macrophages (x-axis).

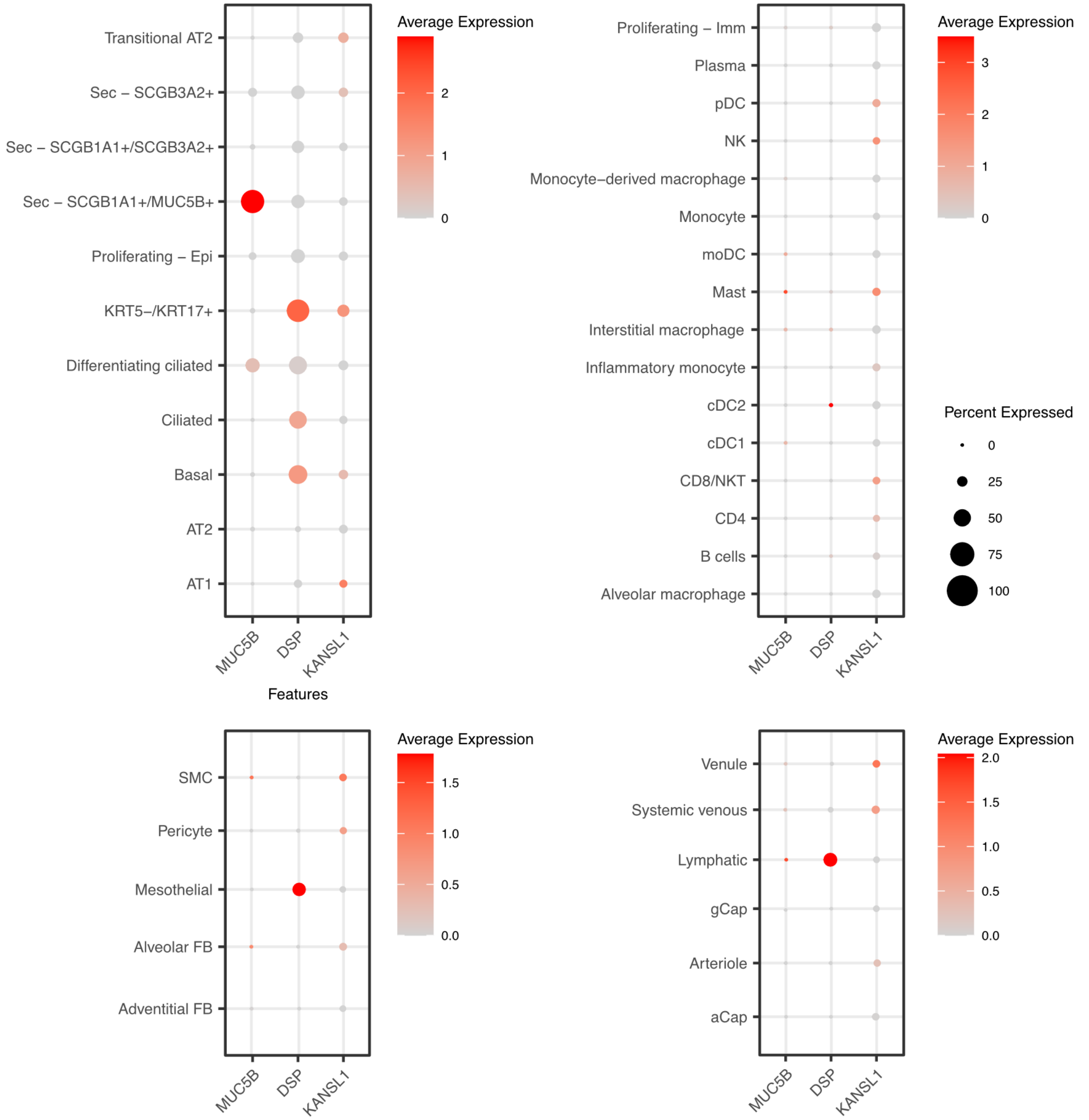

Supplementary Figure 17: Expression of *MUC5B* across tested cell types.

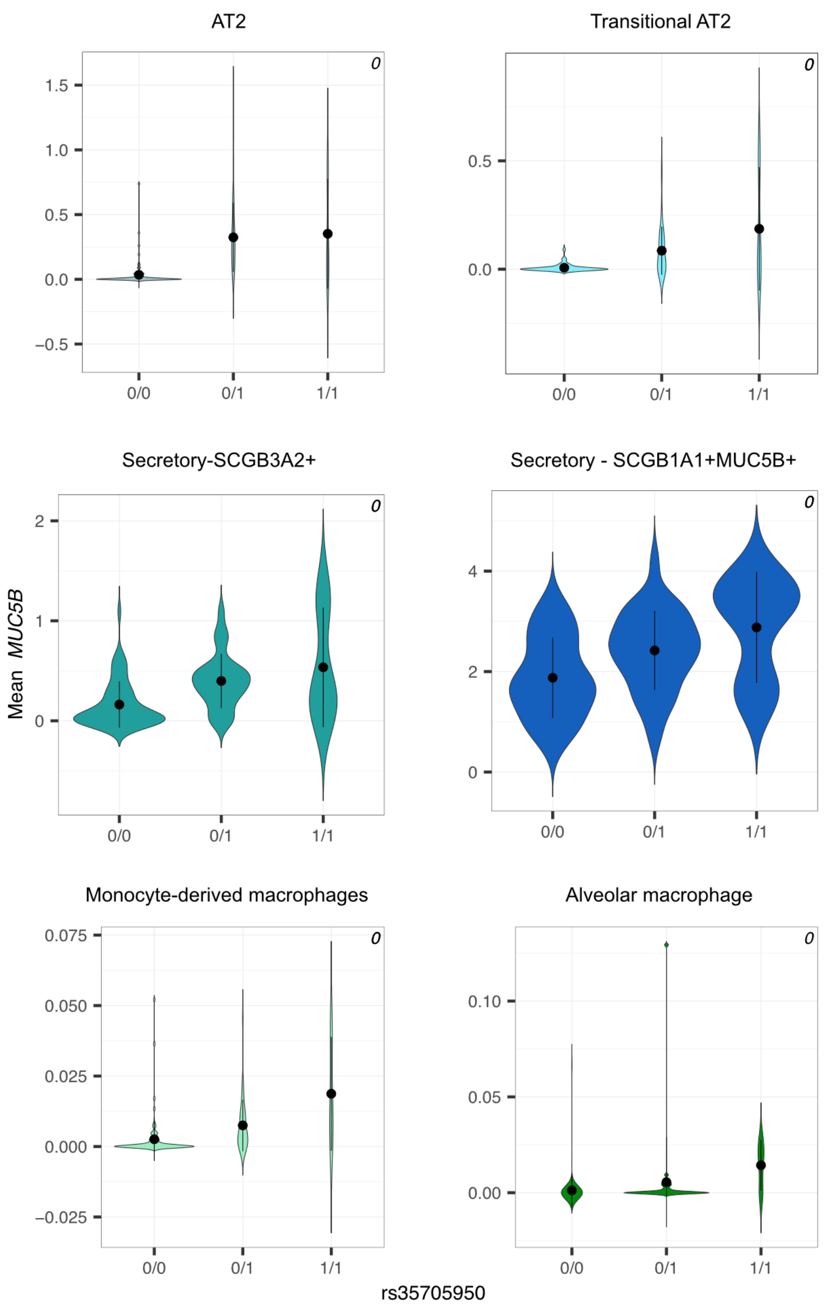

Supplementary Figure 18: eQTL violinplots of the top GWAS variant for *MUC5B* in cell types in which significant colocalization with GWAS was detected. Mean and two SDs are indicated. 0 indicates the reference allele; 1 indicates the alternative allele (0/0 n=70, 0/1 n=33, 1/1 n=10).

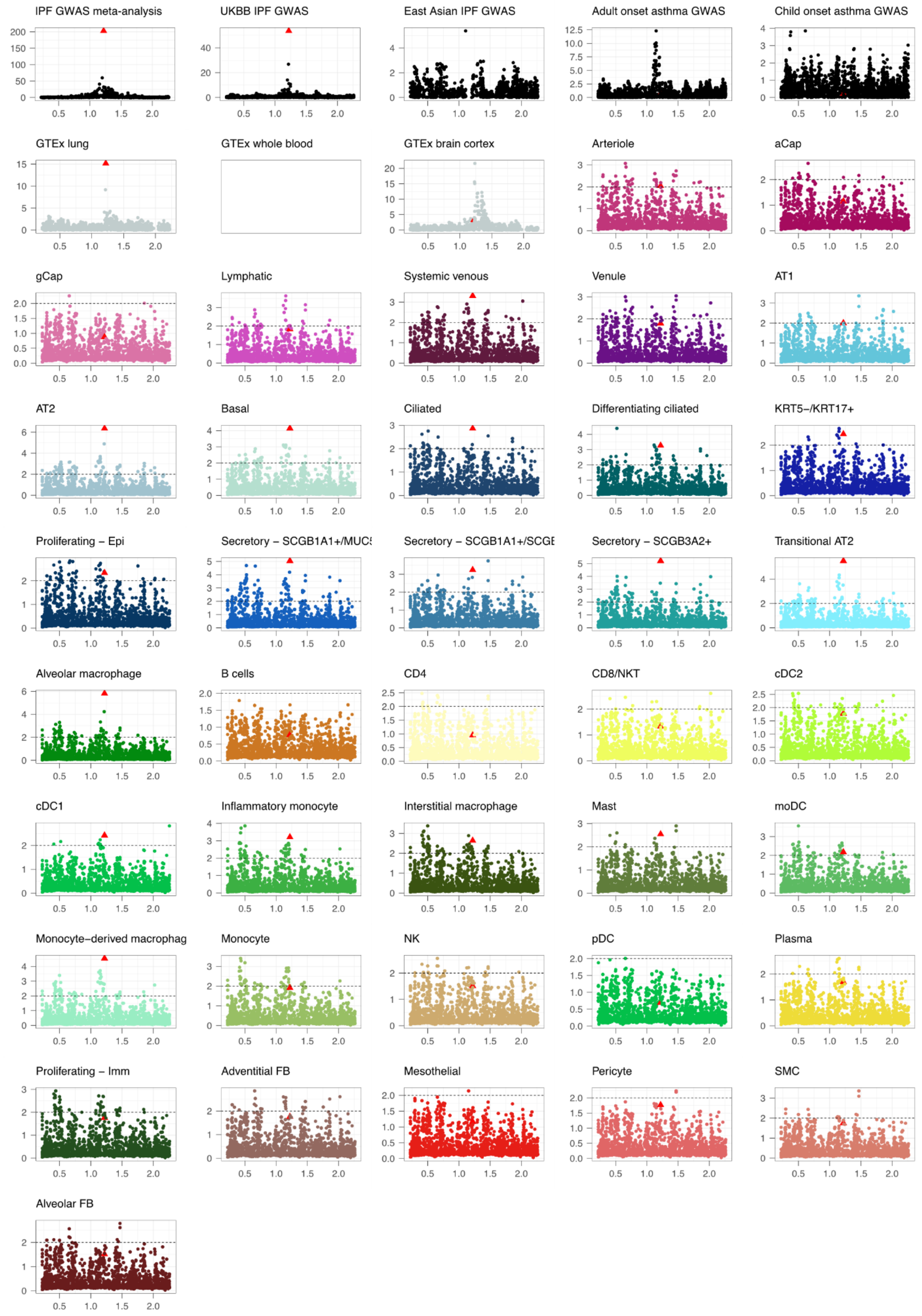

Supplementary Figure 19: Manhattan plots of -$\log_{10}$ nominal *p*-values or mashr lfsr-values (y-axis) for *MUC5B* eQTL and GWAS. Basepare positions are indicated in Mb. IPF GWAS top variant is indicated as a red triangle.

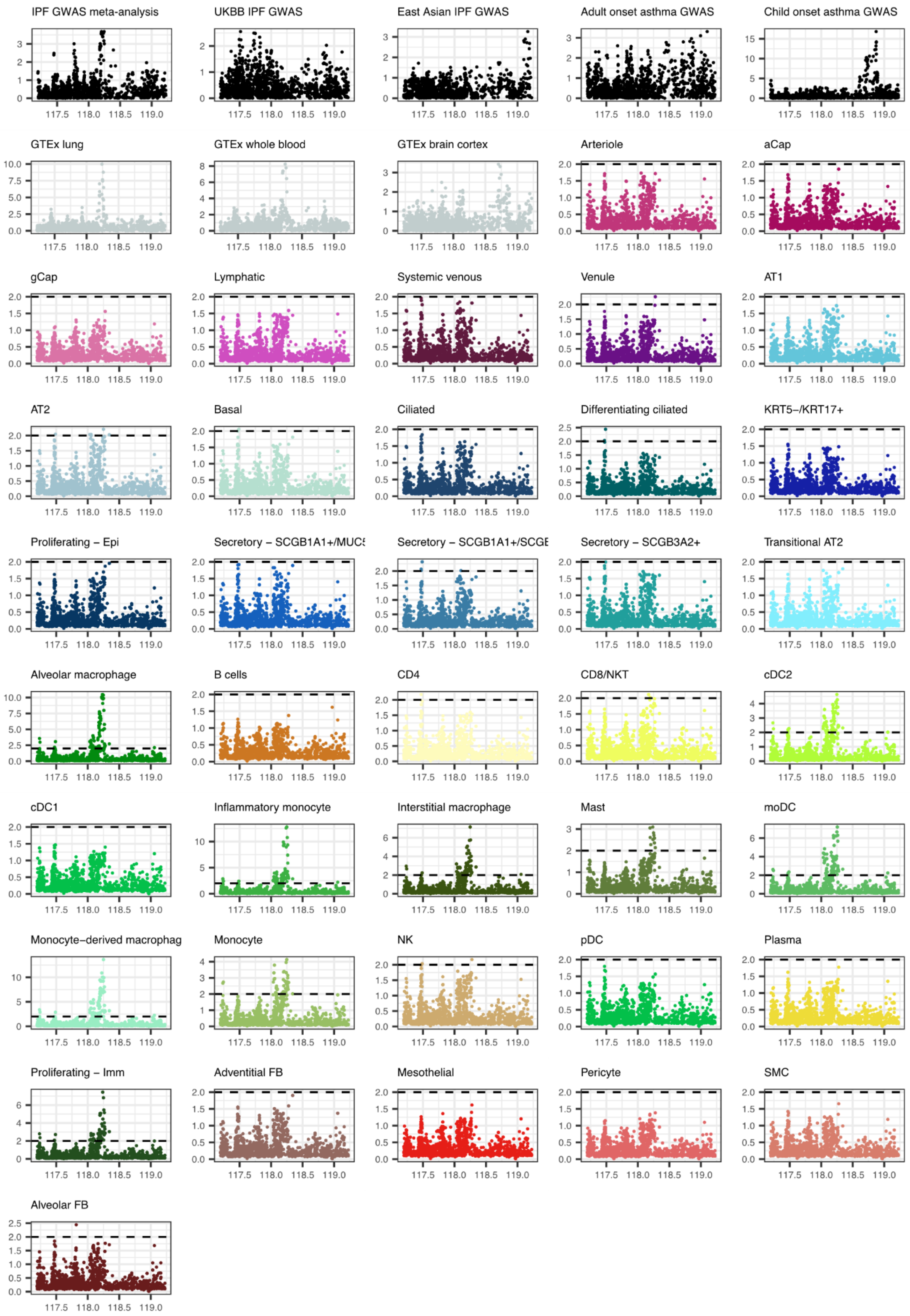

Supplementary Figure 20: Manhattan plots of -$\log_{10}$ nominal *p*-values or mashr lfsr-values (y-axis) for *JAML* eQTL and GWAS. Basepair positions are indicated in Mb.

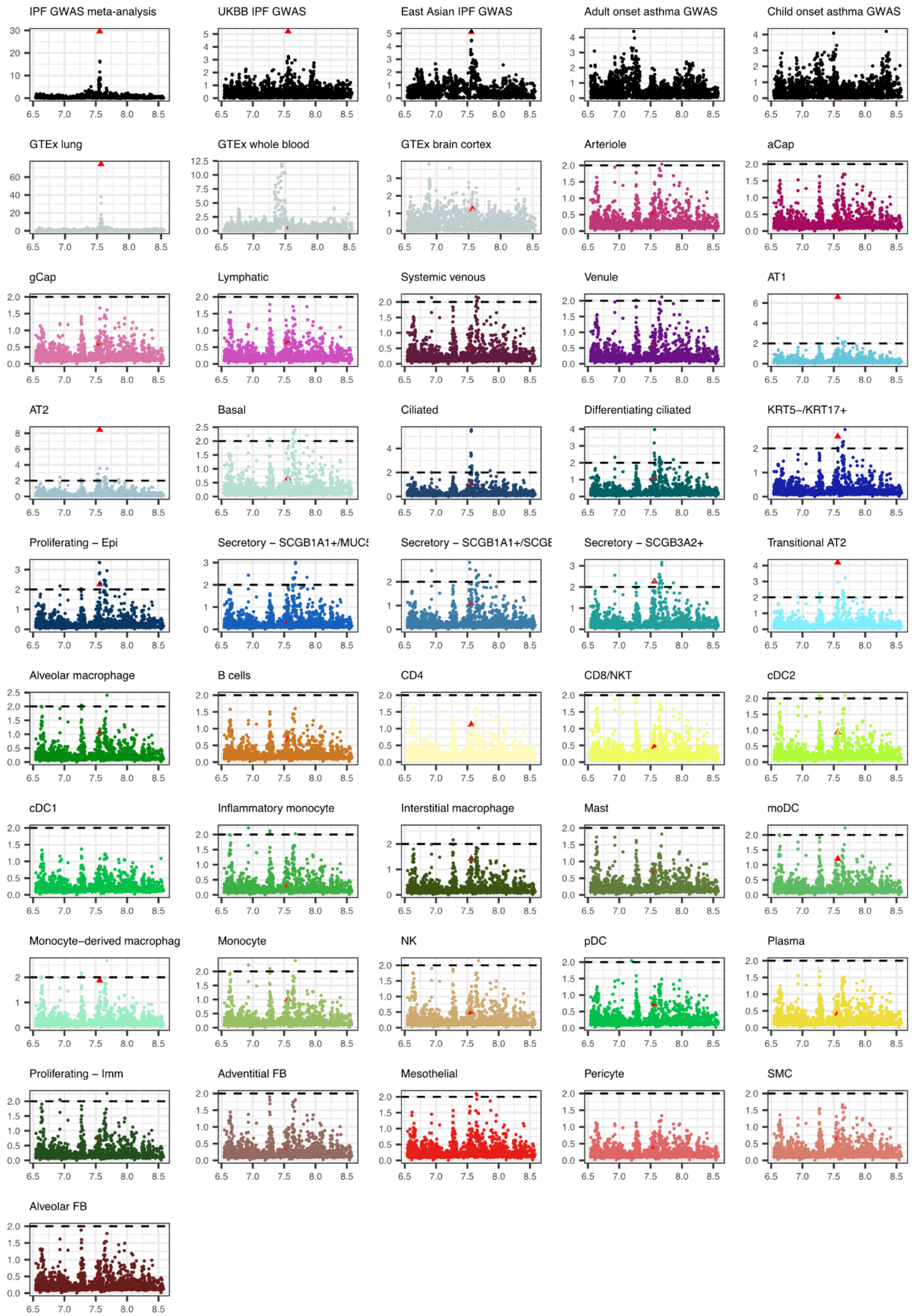

Supplementary Figure 21: Manhattan plots of -$\log_{10}$ nominal *p*-values or mashr lfsr-values (y-axis) for *DSP* eQTL and GWAS. Basepare positions are indicated in Mb. IPF GWAS top variant is indicated as a red triangle.

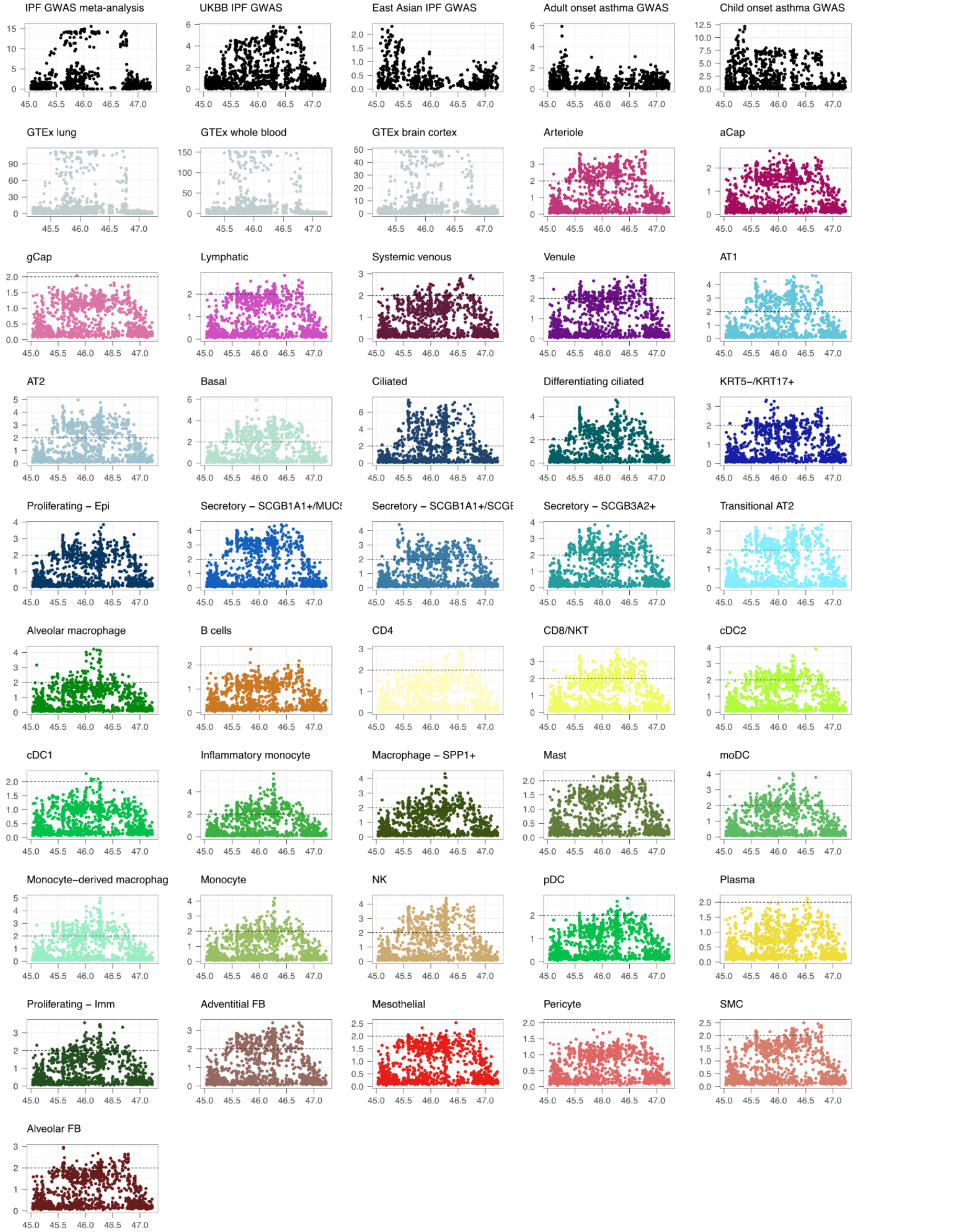

Supplementary Figure 22: Manhattan plots of -$\log_{10}$ nominal *p*-values or mashr lfsr-values (y-axis) for *KANSL1* eQTL and GWAS. Basepair positions are indicated in Mb.

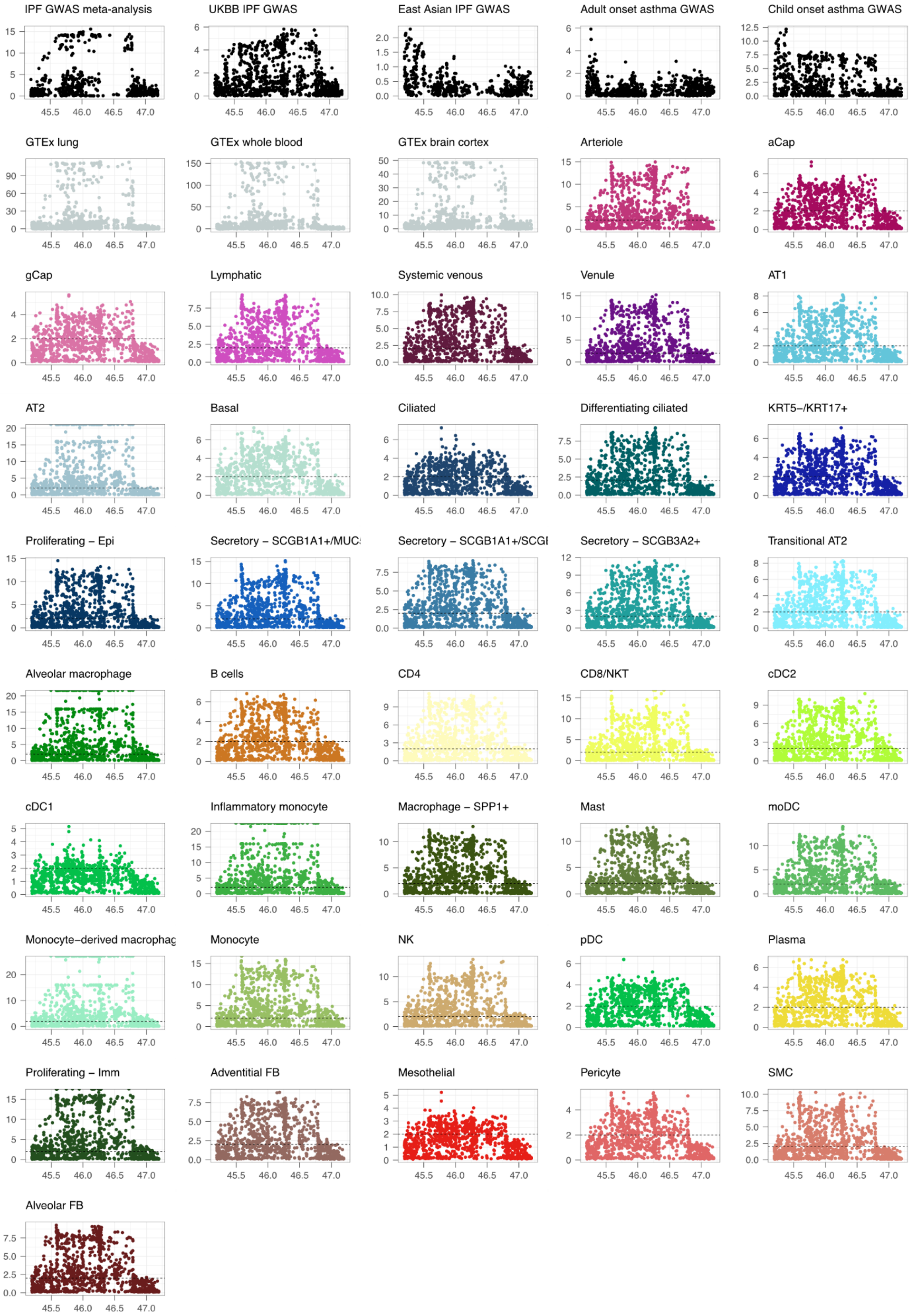

Supplementary Figure 23: Manhattan plots of -$\log_{10}$ nominal *p*-values or mashr lfsr-values (y-axis) for *KANSL1-AS1* eQTL and GWAS. Basepair positions are indicated in Mb.

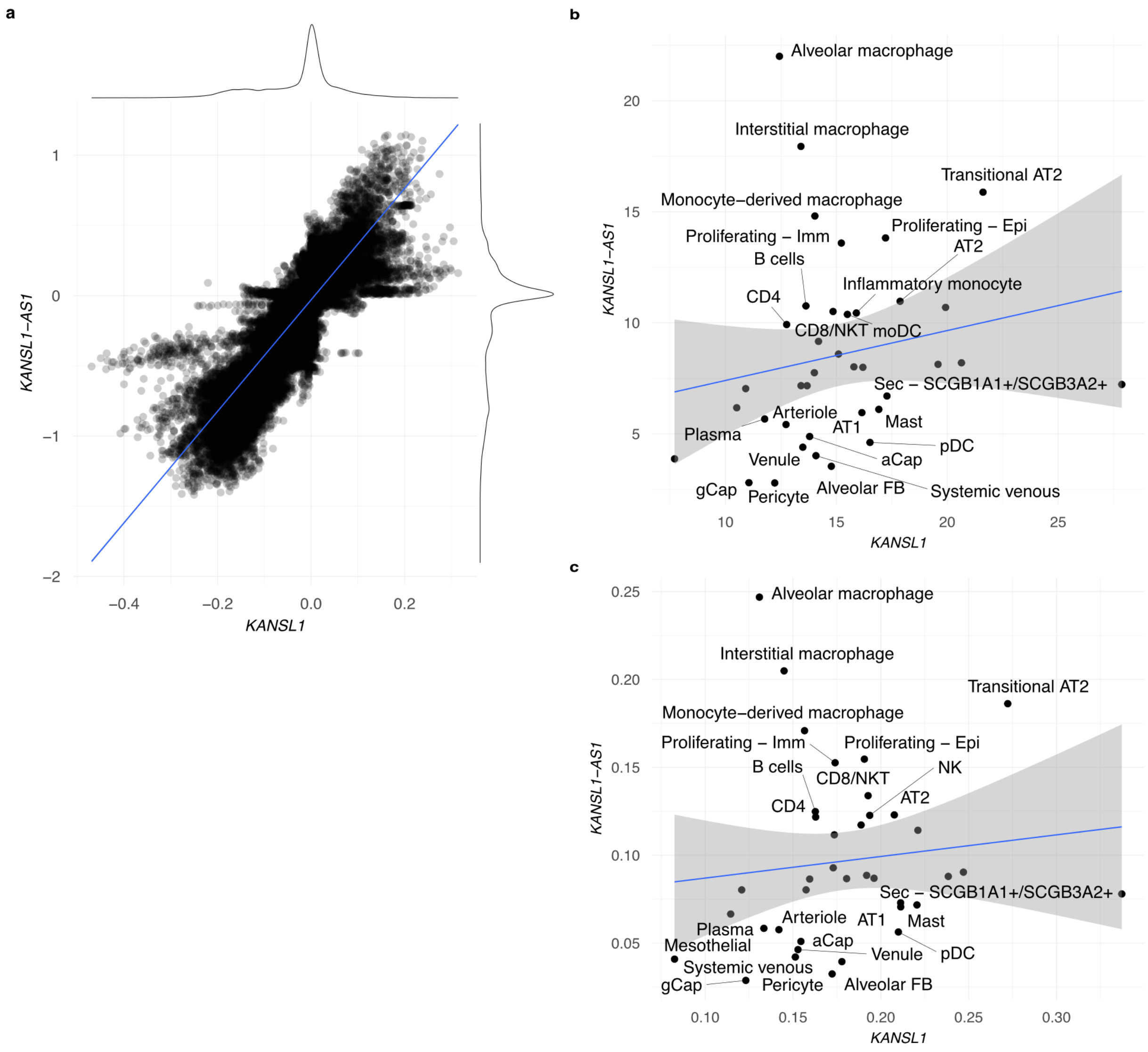

Supplementary Figure 24: a, *KANSL1* and *KANSL1-AS1* eQTL mashr posteriors. **b,** Proportion of cells expressing and **c,** average expression of *KANSL1* and *KANSL1-AS1* in tested cell types. Outliers are labeled and a 95% confidence interval is indicated.

References

1. Cuomo, A. S. E. *et al.* Optimizing expression quantitative trait locus mapping workflows for single-cell studies. *Genome Biol.* **22**, 188 (2021).
2. Yazar, S. *et al.* Single-cell eQTL mapping identifies cell type-specific genetic control of autoimmune disease. *Science* **376**, eabf3041 (2022).
