# Supplementary material for: Cell-type-specific and disease-associated expression quantitative trait loci in the human lung
Source: Nat Genet. 2024 Mar 28;56(4):595–604. doi: 10.1038/s41588-024-01702-0 (PMC11018522; doi:10.1038/s41588-024-01702-0)
Supplement: Supplementary file 2 — Reporting Summary [file 41588_2024_1702_MOESM2_ESM.pdf]

nature portfolio

Corresponding author(s): Nicholas E Banovich
nbanovich@tgen.org

Last updated by author(s): Oct 6, 2023

Reporting Summary

Nature Portfolio wishes to improve the reproducibility of the work that we publish. This form provides structure for consistency and transparency in reporting. For further information on Nature Portfolio policies, see our Editorial Policies and the Editorial Policy Checklist.

Statistics

For all statistical analyses, confirm that the following items are present in the figure legend, table legend, main text, or Methods section.

n/a	Confirmed	
☐	☒	The exact sample size (*n*) for each experimental group/condition, given as a discrete number and unit of measurement
☐	☒	A statement on whether measurements were taken from distinct samples or whether the same sample was measured repeatedly
☐	☒	The statistical test(s) used AND whether they are one- or two-sided<br>*Only common tests should be described solely by name; describe more complex techniques in the Methods section.*
☐	☒	A description of all covariates tested
☐	☒	A description of any assumptions or corrections, such as tests of normality and adjustment for multiple comparisons
☐	☒	A full description of the statistical parameters including central tendency (e.g. means) or other basic estimates (e.g. regression coefficient) AND variation (e.g. standard deviation) or associated estimates of uncertainty (e.g. confidence intervals)
☐	☒	For null hypothesis testing, the test statistic (e.g. *F*, *t*, *r*) with confidence intervals, effect sizes, degrees of freedom and *P* value noted<br>*Give P values as exact values whenever suitable.*
☐	☒	For Bayesian analysis, information on the choice of priors and Markov chain Monte Carlo settings
☐	☒	For hierarchical and complex designs, identification of the appropriate level for tests and full reporting of outcomes
☐	☒	Estimates of effect sizes (e.g. Cohen's *d*, Pearson's *r*), indicating how they were calculated

Our web collection on statistics for biologists contains articles on many of the points above.

Software and code

Policy information about availability of computer code

Data collection

Provide a description of all commercial, open source and custom code used to collect the data in this study, specifying the version used OR state that no software was used.

Data analysis

Publicly available software used in data analysis:
CellRanger Count v3.0.2
R/Seurat v4
plink2 v1.07
LIMIX (https://github.com/single-cell-genetics/LIMIX_qtl)
R/mashr v0.2
R/ashr v2.2
R/coloc v5
R/TopGO v2.46.0
R/nullranges v3.16
HOMER v4.11

Custom scripts to reproduce the result presented here are available on GitHub at https://github.com/tgen/banovichlab/tree/master/ILD_eQTL.

For manuscripts utilizing custom algorithms or software that are central to the research but not yet described in published literature, software must be made available to editors and reviewers. We strongly encourage code deposition in a community repository (e.g. GitHub). See the Nature Portfolio guidelines for submitting code & software for further information.

Data

Policy information about availability of data

All manuscripts must include a data availability statement. This statement should provide the following information, where applicable:

- Accession codes, unique identifiers, or web links for publicly available datasets
- A description of any restrictions on data availability
- For clinical datasets or third party data, please ensure that the statement adheres to our policy

Raw and processed 10x Genomics data, Seurat objects, mean-aggregated expression matrices, and genome-wide LIMIX and mashr eQTL statistics can be found on GEO with the accession number GSE227136. Genotype data are available on dbGaP with the accession number phs003521.

Human research participants

Policy information about studies involving human research participants and Sex and Gender in Research.

Reporting on sex and gender	Self-reported gender information was available for 107 out of the 116 donors. Out of these, 29 reported female and 78 reported male. No sex-specific analyses were performed in this study due to the low number of female samples.
Population characteristics	Data were collected from 114 individuals, including 66 (58%) with ILD and 48 (42%) unaffected donors. The ILD lungs included samples from 39 individuals with IPF and 27 with other forms of PF, including sarcoidosis (n=4), connective tissue disease-associated interstitial lung disease (CTD-ILD, n=3), idiopathic nonspecific interstitial pneumonia (NSIP, n=3), coal worker's pneumoconiosis (CWP, n=3), chronic hypersensitivity pneumonitis (cHP, n=2), interstitial pneumonia with autoimmune features (IPAF, n=2), and unclassifiable ILD (n=10). The majority (67%) of the lung samples were from individuals with self-reported ethnicity information of European ancestry, and 53 (46%) reported past or present tobacco use.
Recruitment	Participants were be patients of the Clinical Investigator that are scheduled for a lung transplant surgery. The Clinical Investigator or a member of his research staff approached individuals to discuss the study and invite them to participate.
Ethics oversight	Studies were approved by the local Institutional Review Boards (Vanderbilt IRB nos. 060165 and 171657 and Western IRB no. 20181836).

Note that full information on the approval of the study protocol must also be provided in the manuscript.

Field-specific reporting

Please select the one below that is the best fit for your research. If you are not sure, read the appropriate sections before making your selection.

☒ Life sciences ☐ Behavioural & social sciences ☐ Ecological, evolutionary & environmental sciences

For a reference copy of the document with all sections, see nature.com/documents/nr-reporting-summary-flat.pdf

Life sciences study design

All studies must disclose on these points even when the disclosure is negative.

Sample size	The final dataset consisted of data collected from 114 donors, including 66 ILD and 48 unaffected donors.
Data exclusions	Donor VUILD65 was removed due to inconsistencies in metadata suggesting mislabeling.
Replication	sc-eQTL were compared with previously published datasets, i.e., GTEx.
Randomization	This is not relevant to the present study, as samples were not allocated to groups but comparisons were conducted between cases and unaffected donors.
Blinding	This is not relevant to the present study as samples were not allocated to groups.

Reporting for specific materials, systems and methods

We require information from authors about some types of materials, experimental systems and methods used in many studies. Here, indicate whether each material, system or method listed is relevant to your study. If you are not sure if a list item applies to your research, read the appropriate section before selecting a response.

Materials & experimental systems

n/a	Involved in the study
☒	☐ Antibodies
☒	☐ Eukaryotic cell lines
☒	☐ Palaeontology and archaeology
☒	☐ Animals and other organisms
☒	☐ Clinical data
☒	☐ Dual use research of concern

Methods

n/a	Involved in the study
☒	☐ ChIP-seq
☒	☐ Flow cytometry
☒	☐ MRI-based neuroimaging
